# LC–MS/MS, GC–MS and molecular docking analysis for phytochemical fingerprint and bioactivity of *Beta vulgaris* L.

**DOI:** 10.1038/s41598-024-58338-7

**Published:** 2024-03-29

**Authors:** Özge Üst, Emine Yalçin, Kültiğin Çavuşoğlu, Burak Özkan

**Affiliations:** 1 Department of Biology, Institute of Science, Giresun University, Giresun, Turkey; 2 Department of Biology, Faculty of Science and Art, Giresun University, Giresun, Turkey

**Keywords:** *Beta vulgaris*, Bioactivity, LC–MS/MS, GC–MS, Molecular docking, Phytochemicals, Molecular biology, Plant sciences

## Abstract

The plants that we consume in our daily diet and use as a risk preventer against many diseases have many biological and pharmacological activities. In this study, the phytochemical fingerprint and biological activities of *Beta vulgaris* L. leaf extract, which are widely consumed in the Black Sea region, were investigated. The leaf parts of the plant were dried in an oven at 35 °C and then ground into powder. The main constituents in *B. vulgaris* were identified by LC–MS/MS and GC–MS analyses. Phenolic content, betaxanthin and betacyanin levels were investigated in the extracts obtained using three different solvents. The biological activity of the extract was investigated by anti-microbial, anti-mutagenic, anti-proliferative and anti-diabetic activity tests. Anti-diabetic activity was investigated by in vitro enzyme inhibition and in-silico molecular docking was performed to confirm this activity. In the LC–MS analysis of *B. vulgaris* extract, a major proportion of p_coumaric acid, vannilin, protecatechuic aldehyde and sesamol were detected, while the major essential oils determined by GC–MS analysis were hexahydrofarnesyl acetone and phytol. Among the solvents used, the highest extraction efficiency of 2.4% was obtained in methanol extraction, and 36.2 mg of GAE/g phenolic substance, 5.1 mg/L betacyanin and 4.05 mg/L betaxanthin were determined in the methanol extract. *Beta vulgaris*, which exhibited broad-spectrum anti-microbial activity by forming a zone of inhibition against all tested bacteria, exhibited anti-mutagenic activity in the range of 35.9–61.8% against various chromosomal abnormalities. *Beta vulgaris* extract, which did not exhibit mutagenic, sub-lethal or lethal effects, exhibited anti-proliferative activity by reducing proliferation in *Allium* root tip cells by 21.7%. 50 mg/mL *B. vulgaris* extract caused 58.9% and 55.9% inhibition of α-amylase and α-glucosidase activity, respectively. The interactions of coumaric acid, vanniline, hexahydrofarnesyl acetone and phytol, which are major compounds in phytochemical content, with α-amylase and α-glucosidase were investigated by in silico molecular docking and interactions between molecules via various amino acids were determined. Binding energies between the tested compounds and α-amylase were obtained in the range of − 4.3 kcal/mol and − 6.1 kcal/mol, while for α-glucosidase it was obtained in the range of − 3.7 kcal/mol and − 5.7 kcal/mol. The biological activities of *B. vulgaris* are closely related to the active compounds it contains, and therefore studies investigating the phytochemical contents of plants are very important. Safe and non-toxic plant extracts can help reduce the risk of various diseases, such as diabetes, and serve as an alternative or complement to current pharmaceutical practices.

## Introduction

Food is the most important prerequisite for the survival of any organism, and plants are the most basic source of food, as they meet most of the human needs. Wild plants have been used as food throughout history, and some plant species are also used in various industrial areas such as medicine, cosmetics and textiles. Interest in the consumption of plant foods is increasing day by day due to their potential as alternative disease treatments, reducing the risk of diseases and reducing health care costs. Natural medicinal plants and preparations derived from plants represent an important alternative source for the discovery of new drugs and existing synthetic drugs. Approximately a quarter of the current medicines used in the healthcare industry are of plant origin, representing only 5–15% of the medicinal herbs researched. The rich plant diversity in the world shows that there is still a large gap in drug discovery from herbal sources^1-4^. Plants synthesize and accumulate in their tissues a large number of complex secondary metabolites that reduce the incidence of certain diseases and have health-promoting properties^5,6^. Many secondary compounds do not have a role in plant development and main metabolism, but are involved in defense against various environmental stress factors such as pathogens, radiation and heat stress. Secondary compounds that provide protection against stress conditions have many biological and pharmacological properties^7^. Although there are many studies in the literature investigating plant secondary compounds and phytochemical compounds, the richness of plant diversity makes these studies insufficient. In this study, the phytochemical fingerprint and biological activities of *B. vulgaris* L. collected from Giresun–Bulancak region were investigated. *Beta vulgaris* (beet) is a flowering plant species belonging to the Betoideae subfamily of the Amaranthaceae family. *Beta vulgaris* can be distributed in northern and southeastern Europe, Atlantic coasts, Mediterranean, North Africa, Western Asia and regions with temperatures between 15 and 19 °C. *Beta vulgaris* mainly consists of root and leaf parts. The sugar formed as a result of photosynthesis in the plant is stored in the root in the form of a tuber. Tubers are commercially used in sugar production because they contain high concentrations of sucrose and the leaves are consumed as food in many countries. *Beta vulgaris* also contains strong anti-oxidant compounds such as triterpene, sesquiterpenoid, carotenoid, coumarin, flavonoid (tiliroside, astragalin, rhamnocitrine, ramnetin, and kaempferol), betalains and phenolic compounds^8^. *Beta vulgaris* extracts with rich betalain content exhibit anti-microbial activity by chelating internal cations necessary for the cell in microorganisms, and anti-malarial activity by blocking the transport of choline into the cell in parasites^9^. Betalain compounds also contribute to the anti-carcinogenic effect of *B. vulgaris* with anti-inflammatory, pro-apoptotic and anti-proliferative mechanisms^10^. The rich phytochemical content detected in plants is responsible for the formation of various biological activities. Among these activities, anti-hyperglycemic activity comes to the forefront due to increasing diabetes cases. Diabetes treatments aim to prevent increases in blood sugar. Plants with anti-hyperglycemic effects have multiple mechanisms of action, such as suppressing blood glucose increases, inducing glucose uptake into tissues, and reducing glucose absorption from the intestines. Among these mechanisms, the anti-diabetic activity of plants is mostly achieved by reducing the digestion of carbohydrates in the diet. Herbal extracts can reduce carbohydrate digestion by inhibition of glucosidase and amylase enzymes^11^.

In literature studies, free radical scavenging, anti-inflammatory, anti-oxidant, anti-viral, anti-microbial and hepatoprotective activities of *B. vulgaris* extracts have been reported^12^. Plants of the same species spreading in different regions produce secondary compounds in different structures and amounts according to the climatic conditions and the stress conditions they encounter, and this causes variability in phytochemical content and biological activity^13^. There are studies in the literature on *B. vulgaris* tuber and leaves collected from different regions. However, there is no detailed study on *B. vulgaris* samples distributed in Giresun (Turkey). In this study, a comprehensive study was carried out using data obtained from phytochemical analysis, in-vitro, in-vivo and in-silico analyses. For phytochemival analysis betalain and total phenolic contents were determined and the main components were identified with LC–MS/MS and GC–MS analyses. The biological activities of the extract were determined by investigating anti-bacterial, anti-fungal, anti-mutagenic, anti-proliferative and anti-hyperglycemic activities. Plant foods consumed in the diet, popularly known as anti-microbial and anti-diabetic, may also exhibit cytotoxic properties. For this reason, the cytotoxic effects of foods that exhibit biological activity and are consumed in the diet should also be examined. In this study, in addition to the anti-microbial and anti-diabetic activity of *B. vulgaris*, anti-mutagenic and anti-proliferative effects were also examined and its cytotoxic properties and also its protective properties against this toxicity were tested.

## Materials and methods

High-purity chemicals used in the analyses were purchased from Sigma (St Louis, MO) and Merck (Germany), while carmine (CAS: 1390–65–4) was purchased from Isolab (Eschau, Germany). *Allium cepa* L. (2n = 16) bulbs purchased from Akdeniz Agriculture (TR-55-K-009228, Turkiye).

### Sample collection and extraction

**Figure 1:**
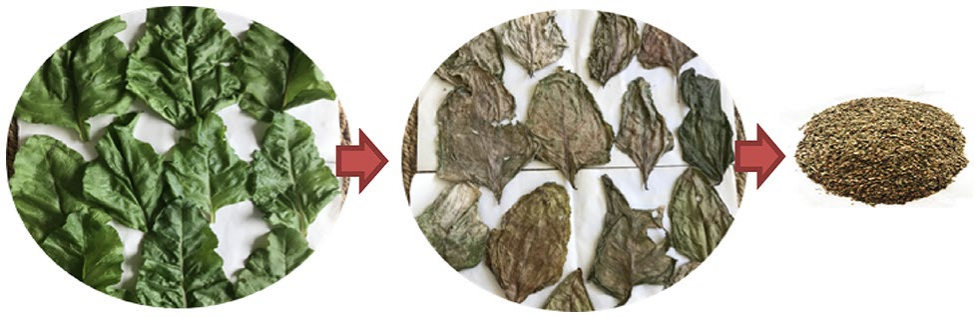
Drying and pulverizing stages of *B. vulgaris* leaves.

*Beta vulgaris* leaves were collected from Giresun-Bulancak region (40.9417459, 38.2868519) in September-2021 and identified by Professor Zafer TÜRKMEN at Giresun University, Department of Botany. A plant specimen bearing the voucher number BIO-Bevu-0105/2021 was preserved in the herbarium. Leaves that were uniform, free of disease, and did not wilt were dried entirely at 35 °C in an oven before being pulverized into a powder using a grinder (Fig. 1). Three distinct solvents were used in the maceration process to extract leaf tissues: methanol, chloroform, and water. After extracting 2 g of leaves in 100 mL of solvent in a shaking incubator at room temperature for 24 h, they were filtered with Whatman No: 4 filter paper. After centrifuging the filtrate for 10 min at 5000 rpm, the supernatant was removed at 40 °C using an evaporator (Hei-VAP, Heidolph, Germany) and the resulting extracts were kept in glass vials at + 4 °C^14^. The extraction efficiency with each solvent was calculated using Eq. (1). Experimental research and field studies on plants, including the collection of plant material, comply with relevant institutional, national, and international guidelines and legislation.

1 $${\text{Extract efficiency }}\left( \% \right):{\text{ Extract obtained after evaporation }}\left( {\text{g}} \right)/{\text{ Amount of plant }}\left( {\text{g}} \right) \, \times { 1}00$$

### Phytochemical analyses

#### Total phenolic content

The Folin–Ciocalteau method was used to calculate the total phenolic content. 200 µL of 10% Na_2_CO_3_, 40 µL of Folin–Ciocalteu reagent, 10 µL of the extract and 10 µL of H_2_O were combined. Following a 30-min incubation period, the mixture’s absorbance at 725 nm was determined. Total phenol content is given as mg GAE/g fresh weight as gallic acid equivalent (GAE) used as standard.

### Quantitative betalain analysis

Betalain analysis in leaf extract was determined by measuring betaxanthin and betacyanin levels. For betalain extraction, 0.5 g lyophilized sample was homogenized in 20 mL of 50% ethanol for 20 min and the homogenate was centrifuged at 6000 rpm for 10 min. The same process was repeated three times for maximum betaxanthin and betacyanin extraction. Supernatants were collected and used for betaxanthin and betacyanin analysis. Betaxanthin and betacyanin contents in the extracts were determined by spectrometric measurement at 538 nm and 480 nm, respectively, according to the method suggested by Stintzing et al.^15^ The total betalain content was calculated by Eq. (2) using the absorbances obtained.

2 $${\text{BX or BC content }}\left( {{\text{mg}}/{\text{L}}} \right) \, = \, \left[ {\left( {{\text{A }} \times {\text{ DF }} \times {\text{ MW }} \times { 1}000} \right) \, / \, \left( {{\text{e }} \times {\text{ l}}} \right)} \right]$$

DF: dilution factor, A: absorption, 1: path length of the cuvette (1 cm). Molecular weight of betacyanin: MW = 550 g/mol, molar extinction coefficients for betacyanin (e) = 60,000 L/mol^−1^ cm^−1^, Molecular weight of betaxanthin: MW = 308 g/mol, molar extinction coefficients for betaxanthin (e) = 48,000 L/mol^−1^ cm^−1^.

### LC–MS/MS

The profile of phenolic compounds in *B. vulgaris* was determined by LC–MS/MS analysis. 1 g of leaf samples was extracted with 4:1 methanol-dichloromethane solvent (4:1). The extract was filtered with a 0.45 μm sterile syringe and the filtrate was used in phenolic substance analysis. ODS Hypersil 4.6*250 mm column was used in the analysis performed on the LC–MS/MS (Thermo Scientific) device. The elution gradient consisted of mobile phase A (water with 0.1% formic acid) and mobile phase B (methanol). The gradient program was fixed as follows: 0–1 min, 0% B; 1–22 min, 95% B; 22–25 min, 95% B; 25–30 min, 100% B. Total time of evaluation was 34 min with conditioning time^16,17^. LC–MS/MS analysis was performed at HUBTUAM-Hitit University.

### Essential oil extraction and GC–MS analyses

The essential oil was obtained from the plant by steam distillation method in the Clevenger apparatus. The samples (35 g) were transferred to the Clevenger flask and 400 mL of water was added. The system was operated continuously for 3 h and the essential oil sample was separated (40 mg), dried over sodium sulfate and stored in the refrigerator. GC–MS analyses were performed with an SQ Quantum XLS mass spectrometer and a Thermo Scientific-Trace GC Ultra system. TG-5MS (30 m × 0.25 mm inner diameter × 0.25 μm film thickness) was used as the capillary column, and 1.0 mL/min high purity Helium (He) was used as the carrier gas. The injection temperature was set at 250 °C. Capillary column was fixed from 50 to 120 °C (rate: 3 °C/min), 120 to 220 °C (rate:3 °C/min, held for 0.67 min), 220 to 250 (rate: 5 °C/ min, held for 5.0 min). Split/non-split (25:1 split) mode was used for 1.0 μL samples (diluted 1/10 in acetone, v/v). Mass spectra of molecules were determined using relative peak areas through WILEY and NIST libraries^18^. GC–MS analyses were performed at Hitit University HUBTUAM.

### Biological activity

The anti-microbial, anti-proliferative, anti-mutagenic and anti-diabetic activities of the extract were investigated. Biological activity analyses were performed with methanol extract, which obtained high efficiency in *B. vulgaris* extraction. The lowest minimum inhibitory concentration (MIC) value obtained against the tested bacteria in anti-microbial activity analyses was determined as 25 mg/mL. The same 25 mg/mL dose was preferred in anti-proliferative and anti-mutagenic activity studies.

### Anti-fungal and anti-bacterial activity

Anti-microbial activity of the extract was determined by MIC and disk diffusion method and tested against *Salmonella enteritidis* ATCC13076, *Escherichia coli* ATCC 25,922, *Bacillus subtilis* IMG 22, *Pseudomonas aeruginosa* ATCC9027, *Staphylococcus aureus* ATCC 25,923, *Bacillus cereus* ATCC 14,579, *Candida albicans* ATCC 90,028 and *Candida tropicalis*. To determine the MIC concentration, bacteria were incubated in Mueller Hinton Broth medium at 37 °C, and a 10^8^ cells/mL (0.5 McFarland) sample from the culture was transferred into 5 mL physiological saline. The fungi were first incubated in Potato Dextrose Broth for 48 h and a sample of 10^6^ CFU/mL (0.5 McFarland) was placed in 5 mL of physiological saline. Methanol extract of *B. vulgaris* at concentrations of 5, 10 25, 50, 75, 100 and 150 mg/mL was added to 200 µL of culture taken from physiological saline. Solutions containing microorganisms and extracts were incubated at 37 °C for 24 h. 100 µL samples from each culture medium were homogeneously plated onto agar plates and mediums were incubated at 37 °C for 24 h^19^. The lowest concentration without growth was considered the MIC. All experiments were performed three times.

### Anti-mutagenic activity

The anti-mutagenic activity of *B. vulgaris* was determined by the *Allium* test and four different experimental groups were established for this purpose. In Group I, which was considered as negative control, bulbs were germinated with distilled water^20^. Group II, considered as a positive control, was treated with mutagenic sodium azide (NaN_3_). Group III was germinated with only 25 mg/mL methanol extract and the potential mutagenic effect of the extract was investigated in this group. 25 mg/mL NaN_3_ + 25 mg/mL extract was applied to Group IV, and anti-mutagenic activity was determined by calculating the decrease in abnormalities caused by NaN_3_ (Eqs. 3 and 4).

3 $${\text{CA }}\left( \% \right) \, = {\text{A}}/{\text{B }} \times {1}00$$

4 $${\text{Anti}} - {\text{mutagenic activity }}\left( \% \right) \, = \, \left[ {\left( {{\text{a}} - {\text{b}}} \right)/\left( {{\text{a}} - {\text{c}}} \right)} \right] \, \times { 1}00$$

A: total number of abnormal chromosomes, B: total number of cells, a: %CA of the NaN_3_ applied group, b: %CA of the extract + NaN_3_ applied group, c: %CA of the control group.

### Anti-proliferative activity

Four different groups were established to determine the effect of the extract on cell proliferation. The control group was treated with distilled water, the positive control group was treated with 25 mg/mL Glyphosate, and the application group was treated with 25 mg/mL extract. At the end of the application period, root slides were prepared and dividing cells were detected^21^. 5000 cells were counted in each group and the MI percentage was calculated using Eq. (5).

5 $${\text{MI }}\% \, = {\text{ Number of dividing cells}}/{\text{total number of cells }} \times { 1}00$$

To prepare slides from the root samples, 1 cm long root tips collected from each group were fixed with Clarke’s solution. After incubation in serial ethanol solutions, root tips hydrolyzed in 1N HCl were stained with acetocarmine and examined with an advanced research microscope^22^.

### Anti-diabetic activity

Anti-diabetic activity of *B. vulgaris* extract was determined by investigating α-amylase and α-glucosidase inhibition activities. For α-amylase inhibition assay, the method suggested by Nickavar et al.^23^ was used. For α-glucosidase inhibition assay**,** the method suggested by Fang et al.^24^ was used. All analyzes were conducted in triplicate.

### Molecular docking of α-amylase and α-glucosidase

Enzyme inhibitions tested in vitro were confirmed in-silico molecular docking. In this context, the interactions of α-amylase and α-glucosidase enzymes with major compounds in the extract content were investigated and the anti-diabetic activity was confirmed. 3D structures of α-amylase (RDB ID:5KZW)^25^ and α-glucosidase (RDB ID:5KZW)^26^ were retrieved from the Protein Data Bank (PDB, http://www.rcsb.org.pdb). Ligand molecules as phytol, vanilline, p_coumaric acid and hexahydrofarnesyl acetone were edited with Avogadro program after obtaining from PubChem (https://pubchem.ncbi.nlm.nih.gov/compound/). The macromolecules were arranged with the help of the Schrödinger Maestro program. Docking was done with Autodock Vina version 4.2.6. Layout analysis and 3D visualizations were made with Biovia Discovery Studio 2020 Client.

### Statistical analysis

Analyzes were carried out with the "IBM SPSS Statistics 22" program. Experimental results are given as mean ± SD (standard deviation). Statistical significance between the data was determined by Duncan test and One-way ANOVA, and *p* value < 0.05 is statistically significant.

## Results and discussion

In this study, *B. vulgaris* leaves, which are consumed as food in the Black Sea region, especially in Giresun, were extracted, a detailed phytochemical analysis was carried out and various biological activities were investigated.

### Extraction efficiency, betacyanin, betaxanthin and phenolic contents

**Figure 2:**
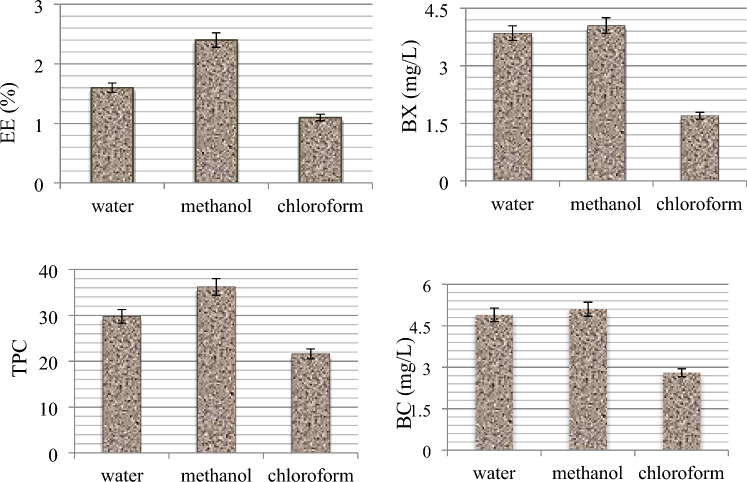
Extraction efficiency and phytochemical content obtained with different solvents. EE: extraction efficiency, TPC: Total phenolic content (mg GAE/g), BX: betaxanthin (mg/L), BC: betacyanin (mg/L).

The extraction efficiency, betacyanin, betaxanthin and total phenolic content of *B. vulgaris* leaves are given in Fig. 2. Among the solvents used in extraction, the highest extraction efficiency was obtained with methanol with a rate of 2.4%. The highest total phenolic substance content in leaf samples was determined as 36.2 mg GAE/g in the methanol extract. Within the scope of the study, the amounts of betacyanin and betaxanthin specific to *B. vulgaris* were also analyzed. Betacyanin content was higher in all extracts compared to betaxanthin. While similar levels of betacyanin and betaxanthin were obtained in water and methanol extracts, lower betalain content was found in chloroform extract compared to other extracts. Betacyanin and betaxanthin contents in methanol extract were found as 5.1 mg/L and 4.05 mg/L, respectively. In the water extract, betacyanin and betaxanthin contents were determined as 4.9 mg/L and 3.85 mg/L, respectively. The methanol extract was used in other analyses as the efficiency and phytochemical content were determined at higher levels in methanol extraction compared to water and chloroform extraction. There are differences in phenolic compound levels of *B. vulgaris* leaves reported in literature studies. Gawlik-Dziki et al.^27^ determined the presence of phenolic content in the range of 8.15–16.55 mg GAE/g and betalain in the range of 2.55–46.42 mg/kg in the methanol/water extracts of *B. vulgaris* leaves. Hajihosseini and Setorki^28^ determined the total phenolic content in ethanol extracts of *B. vulgaris* leaves as 51 mg/g. This variability observed in literature studies can be explained by the use of *B.vulgaris* species grown in different ecological conditions.

### LC–MS/MS analyses

**Figure 3:**
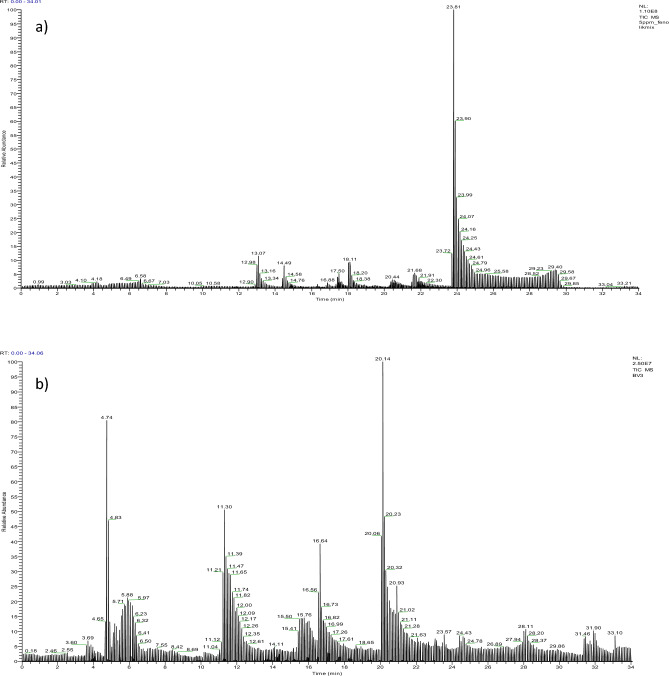
LC–MS/MS chromatograms. Standard compounds (**a**), *B. vulgaris* extract (**b**).

LC–MS/MS results and chromatograms are given in Table 1 and Fig. 3. The chromatogram created using a total of 22 standard substances is given in Fig. 3a. The presence of active compounds such as gallic acid, catechin, rutin, and salicylic acid, which are common phenolic compounds in plants, could not be detected in *B. vulgaris*. Among the tested standards, only the presence of protecatechuic aldehyde, sesamol, vannilin and p_coumaric acid were detected. The highest rate of p_coumaric acid (8.9 mg phenolic/kg) was found in *B. vulgaris* extract, followed by vannilin, protecatechuic aldehyde and sesamol, respectively (Fig. 3b). The identified active compounds make a significant contribution to the biological activity of the plant. Literature studies investigating the phytochemical content of *B. vulgaris* are mostly related to tubers consumed as food. Studies on the analysis of commonly consumed leaf parts are not sufficient. Gawlik-Dziki et al.^27^ reported that they did not detect the content of p-coumaric acid, vanillic acid and caffeic acid in the leaves of *B. vulgaris* grown in Poland, but they detected the presence of high levels of salicylic acid and sinapic acid. Lasta et al.^29^ determined that the *B. vulgaris* leaf extract obtained by ethanol: water extraction contains vanillic acid and chlorogenic acid, but does not contain caffeic acid, cinnamic acid, ferulic acid, gallic acid, p-coumaric acid, sinapic acid, and salicylic acid. These differences in the content of *B. vulgaris* reported in the literature studies and in this study are closely related to the conditions of the habitat. Abiotic factors such as climatic conditions, light intensity, soil structure, rainfall amount and humidity among the places where plants of the same species grow affect the production of phytochemicals. Changes in the growing environment may cause the production of different phytochemicals, as well as the production of the same phytochemical at different levels^30,31^.

**Table 1:** The phenolic compounds of *B. vulgaris* and presence rates.

Compound	mg phenolic/kg	Presence rates (%)
Gallic acid	NF	
Protecatechuic acid	NF	
Protecatechuic aldehyde	4.2	
Sesamol	3.3	
Catechin	NF	
epicatechin	NF	
Caffeic acid	NF	
Syringic acid	NF	
Vannilin	7.3	
Taxifolin	NF	
p_coumaric acid	8.9	
Ferulic acid	NF	
4_OH benzoic acid	NF	
Salicylic acid	NF	
Rosmarinic acid	NF	
Oleuropein	NF	
Routine	NF	
Resveratrol	NF	

NF, not found.

### GC–MS analysis

**Figure 4:**
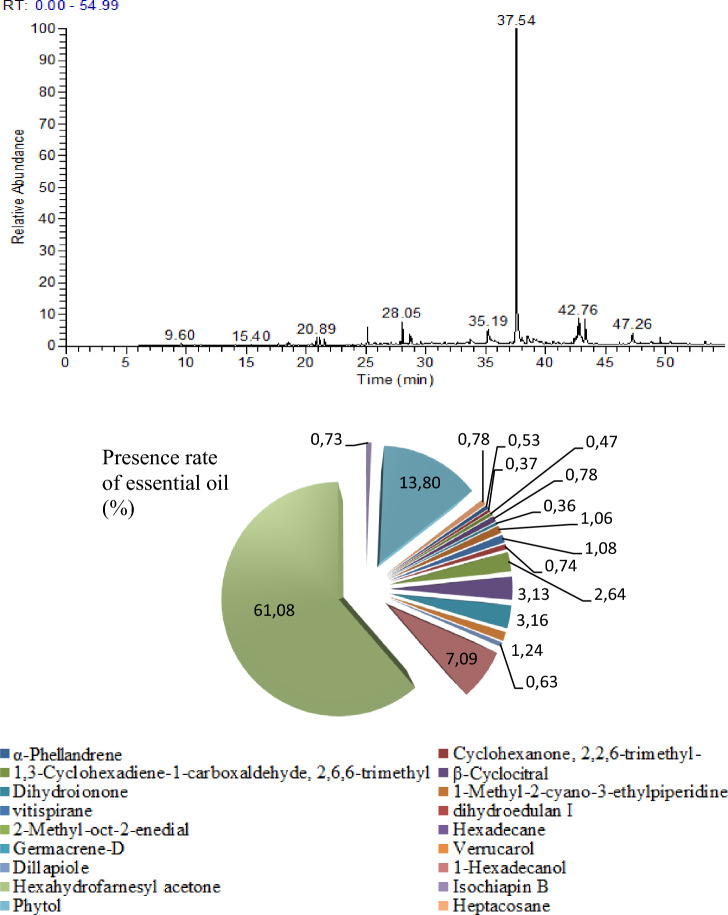
GC–MS chromatogram of *B. vulgaris* and the presence rates of essential oils.

GC–MS chromatogram of *B. vulgaris* leaf extract is given in Fig. 4. Hexahydrofarnesyl acetone (6,10,14-Trimethyl-2-pentadecanone) at a rate of 61.08% and phytol with a rate of 13.80% were defined as major essential oils. The presence rates of hexadecane, germacrene-d and 1-hexadecanol compounds range from 3.13 to 7.09%. Active compounds such as verrucarol, dillapiole, isochiapin b, heptacosane, dihydroedulan I, dihydroionone, vitispirane were also detected and their ratios ranged from 1.24 to 0.36% (Table 2). Essential oils are low molecular weight structures, usually in variable concentrations, and are the main components that make up the biological effects of the plant^32^. Essential oils exhibit strong anti-carcinogenic, anti-microbial anti-proliferative activities. The main mechanisms mediating these activities are the activation of apoptosis and/or necrosis processes and changes in the cell cycle. These mechanisms are closely related to the lipophilic nature of essential oils, which allows them to cross cell membranes, changes in membrane fluidity and composition, leading to leakage of cytoplasmic molecules and ions. These changes in the membrane lead to the changes in pH gradient, decreased ATP production, loss of organel function and cell death^33^. As a cumulative result of all these effects, essential oils and herbal extracts containing these active compounds exhibit strong pharmacological activity. The distribution and availability of essential oil components of *B. vulgaris* leaves vary according to the environment in which it grows and the stress conditions it is exposed to. Onanuga et al.^34^ found that diterpene alcohol and aromatic compounds such as phytol (24.20%), 1,3-dimethylbenzene (14.84%) and neophytadiene (6.13%) are the main compounds in the leaves of *B. vulgaris* grown in Nigeria.

**Table 2:** Essential oil profile of *B. vulgaris.*

Retention time (min)	Essential oil	Area %	Chemical class	Refractive Index
09.61	α-Phellandrene	0.53	Terpenoid	n20/1.474
10.72	Cyclohexanone, 2,2,6-trimethyl-	0.37	Ketone	n20/1.443–1.449
17.71	1,3-Cyclohexadiene-1-carboxaldehyde, 2,6,6-trimethyl-	0.47	Monoterpenoid	n20/1.523
18.50	β-Cyclocitral	0.78	Organic oxide	n20/1.497
20.52	Dihydroionone	0.36	Sesquiterpenoid	n20/1.481
20.89	1-Methyl-2-cyano-3-ethylpiperidine	1.06	Piperidin	n20/1.442
21.15	Vitispirane	1.08	Terpenoid	n20/1.499
21.56	Dihydroedulan I	0.74	Terpenoid	n20/1.462
25.13	2-Methyl-oct-2-enedial	2.64	Aldehyde	n20/1.449–1.459
28.05	Hexadecane	3.13	Alkane	n20/1.434
28.67	Germacrene-d	3.16	Sesquiterpenoid	n20/1.473–1.549
30.48	Verrucarol	1.24	Sesquiterpene	n20/ 1.584
32.64	Dillapiole	0.63	Benzodioxoles	n20/1.530–1.532
35.21	1-Hexadecanol	7.09	Fatty alcohol	n79/1.428
37.53	Hexahydrofarnesyl acetone	61.08	Sesquiterpene	n20/1.445–1.451
41.05	Isochiapin b	0.73	Sesquiterpene	n20/1.443–1.581
42.77	Phytol	13.80	Diterpenoid	n20/1.463

### Anti-microbial activity

**Figure 5:**
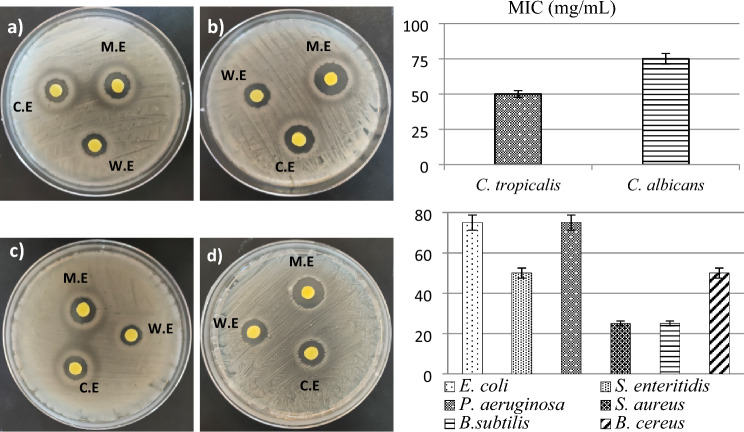
MIC values and maximum inhibition zones of *B. vulgaris* extract. *C. tropicalis* (**a**), *C. albicans* (**b**), *S. enteridis* (**c**), *S. aureus* (**d**). M.E: metanol extract, W.E, water extract; C.E, chlorofom extract.

Inhibition zones used to determine the anti-microbial activity of *B. vulgaris* extract are given in Table 3. Different degrees of inhibition zones were obtained against all tested microorganism species. The fact that the extract forms an inhibition zone against all bacteria indicates that it has a broad spectrum. The highest inhibition zone was obtained against *S. aureus* (18.9 ± 0.3 mm) among gram-positive bacteria and against *S. enteritidis* (15.1 ± 0.6 mm) among gram-negative bacteria. MIC values of *B. vulgaris* extract against *S. enteritidis* and *S. aureus* were determined as 25 mg/mL (Fig. 5). In addition, *B. vulgaris* extract showed a higher cidal effect compared to gram positives than gram negatives. This selectivity of the extract against bacteria can be associated with the structural difference of gram-positive and gram-negative bacteria. Gram-negative bacteria have a double membrane surrounding the cell, and this structure is the main cause of resistance to antimicrobial compounds^35^. Lower inhibition zones were obtained against fungi compared to bacteria. With the application of *B. vulgaris* extract, an inhibition zone of 13.1 ± 0.2 mm against *C. tropicalis* and 14.9 ± 0.4 mm against *C. albicans* were obtained (Fig. 5a,b). *Beta vulgaris* extract showed lower anti-microbial activity against fungi than bacteria. This result can be explained by chitin and ergosterol found in the cell wall structure of fungi. These compounds provide protection against anti-microbials by giving strength to the cell wall*. Candida* species strengthen the development of resistance by increasing the chitin levels in the cell wall when exposed to anti-microbial agents^36^. The anti-microbial activity of *B. vulgaris* extract, which is comparable to standard antibiotics, can be explained by the active ingredients it contains. Phenolic compounds detected by LC–MS/MS analysis and especially essential oils determined by GC–MS analysis have an important effect on the emergence of anti-microbial activity. P-coumaric acid, which was determined as the major compound in LC–MS/MS analysis, has a low MIC value against bacteria. Coumaric acid, which exhibits bactericidal activity by increasing membrane permeability, causing loss of barrier function, leakage of cytoplasmic content and intercalation with the DNA double helix, leads to bacterial death by these mechanisms^37^. Vannilin, the second dominant compound in the extract, similarly disrupts the integrity of the bacterial membrane, causing ions in the cell to leak out, and inhibits intracellular pH homeostasis^38^. Essential oils also exhibit significant anti-microbial activity, and essential oils, which are determined in a high variety in the extract content, are the main factors of the high activity against microorganisms. Hexahydro-farnesyl acetone, which is highly detected in the extract content by GC–MS analysis, has a broad spectrum cidal activity and allelopathic effect against gram-positive, gram-negative bacteria and fungus strains^39^. These effects of hexahydro-farnesyl acetone can be associated with its lipophilic nature, its ability to cross cell membranes and to change membrane composition and membrane fluidity. A strong anti-microbial activity emerges as a result of the cumulative effect of all active ingredients detected in the extract. The anti-microbial activity of *B. vulgaris* leaf extract grown in different ecological environments has also been reported by some literature studies. Ahmadi et al.^40^ reported that the methanol extract of the leaves of *B. vulgaris* distributed in Isfahan (Iran) and the essential oils obtained from the plant with the Clevenger system were effective against bacteria and inhibited mycelial growth in fungi. In the same study, it was stated that the extracts obtained with water were not effective against bacteria.

**Table 3:** Inhibition zones of *B. vulgaris* extract against the tested microorganisms (mm).

Microorganisms	Methanol extract	Water extract	Chloroform	Amikacin	Nistatine
*E. coli*	13.2 ± 0.5^b^	9.9 ± 0.3^d^	11.1 ± 0.2^c^	18.1 ± 0.5^a^	Nt
*S. enteritidis*	15.1 ± 0.6^b^	9.2 ± 0.2^d^	13.5 ± 0.4^c^	16.9 ± 0.6^a^	Nt
*P. aeruginosa*	12.8 ± 0.7^b^	8.6 ± 0.5^d^	10.7 ± 0.1^c^	17.9 ± 0.2^a^	Nt
*S. aureus*	18.9 ± 0.3^b^	13.3 ± 0.5^d^	14.9 ± 0.2^c^	21.2 ± 0.7^a^	Nt
*B.subtilis*	17.6 ± 0.2^b^	11.2 ± 0.7^d^	16.1 ± 0.3^c^	20.9 ± 0.9^a^	Nt
*B. cereus*	15.2 ± 0.8^b^	10.9 ± 0.4^c^	15.1 ± 0.6^b^	18.7 ± 0.3^a^	Nt
*C. tropicalis*	13.1 ± 0.2^b^	10.7 ± 0.5^d^	11.9 ± 0.4^c^	Nt	17.2 ± 0.3^a^
*C. albicans*	14.9 ± 0.4^b^	8.8 ± 0.1^c^	14.8 ± 0.3^b^	Nt	18.1 ± 0.7^a^

Nistatine 30 µg/mL, Amikacin 30 μg/mL.

Nt, not tried. Means shown with different letters^(a–d)^ on the same line are statistically significant (*p* < 0.05).

### Anti-mutagenic activity

**Figure 6:**
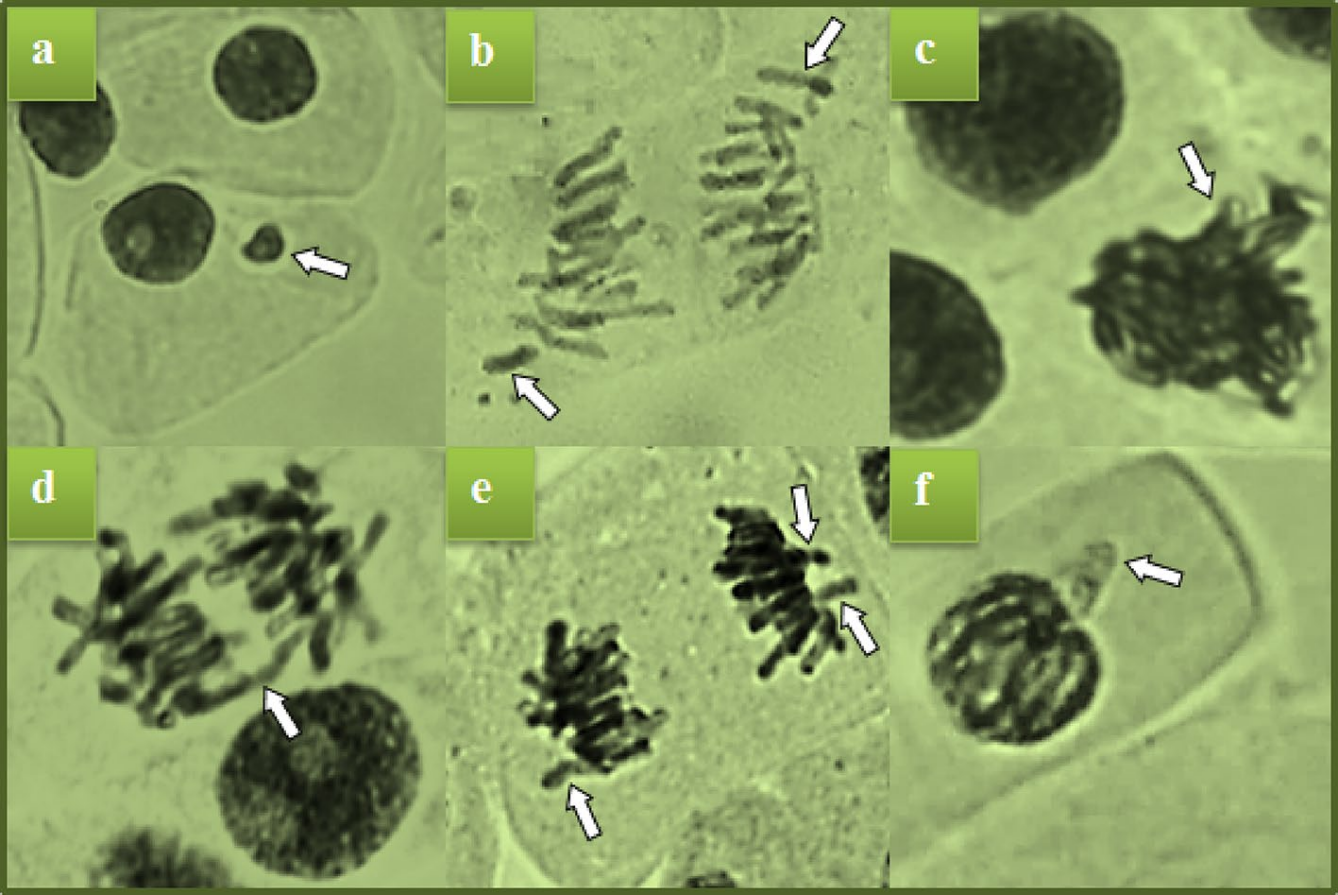
CAs induced by NaN_3._ MN (**a**), fragment (**b**), sticky chromosome (**c**), bridge (**d**), vagrant chromosome (**e**), nuclear bud (**f**).

The anti-mutagenic activity of *B. vulgaris* extract determined by using the *Allium* test is given in Table 4. While a few statistically insignificant fragments and sticky chromosomes were observed in the control group, low levels of MN and vagrant chromosome formation were detected in the only extract-treated group (*p* > 0.05). The statistical insignificant difference between the data of the extract-applied group and the control group indicates that *B. vulgaris* extract does not have a genotoxic effect. High levels and various types of CAs were detected in the positive control group treated with NaN_3_ (Fig. 6). NaN_3_ is a strong mutagen and after the interaction of the azide metabolite with DNA, it has a genotoxic effect by causing mutations, chromosomal irregularities and instabilities^41,42^. The frequency and types of CAs detected in the NaN_3_ group are given in Table 4. The anti-mutagenic activity of the extract was determined by considering the reduction in the frequency of MN and CAs induced by NaN_3_. Anti-mutagenic activity studies were carried out with methanol extract, which achieved high extraction efficiency and a strong anti-microbial effect. The extract, which did not show a genotoxic effect alone, showed an anti-mutagenic effect by reducing the frequency of CAs induced by NaN_3_. *Beta vulgaris* extract exhibited over 50% anti-mutagenic activity against all abnormalities except bridge and nuclear bud formations. The highest anti-mutagenic effect was 61.8% against fragment formations. The extract, which reduced the frequency of MN by 56.4%, provided 56.6% protection against vagrant chromosome formations. It has been reported by many literature studies that the protective effects of herbal products against CAs and MN formations and that this effect is related to the phytochemical content^43–50^. The anti-mutagenic effect of *B. vulgaris* is closely related to the phytochemical components it contains. P-coumaric acid and sesamol are compounds that contribute to this activity. It is known that p-coumaric acid has a wide range of pharmacological effects such as anti-oxidant, anti-microbial, anti-inflammatory, antimutagenic and anti-cancer effects^51^. Sesamol, which exhibits high activity with methylenedioxyphenyl groups in its structure, has anti-oxidant, anti-cancer and anti-mutagenic effects^52^. Essential oils exhibit high cytoprotective properties with synergistic processes and anti-mutagenic effects^53^. Betalains, which have been quantitatively detected in *B. vulgaris* leaves, also exhibit anti-mutagenic activity due to their stability in managing the oxidative stress in the cell^54^. Similar properties of *B. vulgaris* leaves, which exhibit high anti-mutagenic properties with the effect of all active ingredients, have also been reported by literature studies. Klewicka et al.^55^ observed that betalains obtained from *B. vulgaris* were not cytotoxic, but exhibited anti-mutagenic properties against agents with direct mutagenic effects.

**Table 4:** Anti-mutagenic activity of *B. vulgaris* extract against MN and CAs induced by NaN_3_.

	Negative control	Positive control	Methanol extract	Methanol extract + NaN_3_	Anti-mutagenic activity of extract (%)
MN	0.00 ± 0.00^c^	75.6 ± 3.1^a^	0.19 ± 0.42^c^	32.9 ± 2.7^b^	
F	0.29 ± 0.11^c^	61.8 ± 4.9^a^	0.00 ± 0.00^c^	23.6 ± 3.5^b^	
SC	0.45 ± 0.19^c^	52.3 ± 7.5^a^	0.00 ± 0.00^c^	21.9 ± 6.2^b^	
B	0.00 ± 0.00^c^	30.9 ± 6.8^a^	0.00 ± 0.00^c^	19.8 ± 4.1^b^	
VC	0.00 ± 0.00^c^	11.3 ± 2.6^a^	0.22 ± 0.09^c^	4.9 ± 1.9^b^	
NB	0.00 ± 0.00^c^	9.7 ± 2.2^a^	0.00 ± 0.00^c^	5.6 ± 1.1^b^	

1.000 cells were counted in each group for MN and CAs. Means shown with different letters^(a–c)^ on the same line are statistically significant (*p* < 0.05). MN, micronucleus; F, fragment; SC, sticky chromosome; B, bridge; VC, vagrant chromosome; NB, nuclear bud.

### Anti-proliferative activity

The anti-proliferative activity of *B. vulgaris* extract is given in Table 5. In the negative control group, which was considered as control group, a total of 271 cells were in the division phase, but glyphosate application reduced this number to 157. Glyphosate is a compound that delays cell division, causes abnormalities in cell division, and acts at the G_2_/M transition point^56^. Because of these effects on cell proliferation, it is considered a positive control in anti-proliferative assays. Methanol extract of *B. vulgaris* decreased cell proliferation compared to the negative control group. The division rate in cells treated with *B. vulgaris* extract decreased by 21.7% to 212. This decrease in cell proliferation indicates the anti-proliferative effect of *B. vulgaris* extract. Many phenolic compounds have a reducing effect on cell proliferation. Phenolic substances and especially phytol determined in the extract content interact with telomerase in proliferating cells, weakening its activity and exhibiting anti-proliferative effects^57^. Betanin and betaxanthin also exhibit anti-proliferative activity at cell cycle stages^10^. Anti-proliferative activity occurs as a result of the cumulative effect of all compounds detected in *B. vulgaris* extract. Romero et al.^58^ reported that *B. vulgaris* leaf extract obtained from rural areas of Limeira-SP (Brazil) showed anti-proliferative effect by causing accumulation of cells in S and G_0_/G_1_ phases, reduction of cell number in G_2_/M phase and cell cycle arrests.

**Table 5:** Anti-proliferative effects of *B. vulgaris* extract.

	Dividing cell number*	Cell number in interphases	MI (%)
Negative control	271	4729	5.42^a^
25 mg/mL Glyphosate (Positive control)	157	4843	3.14^c^
25 mg/mL methanol extract	212	4788	4.24^b^

*Shows the number of cells in prophase, metaphase, anaphase and telophase. Means shown with different letters^(a–c)^ are statistically significant (*p* < 0.05).

### Anti-diabetic activity

**Figure 7:**
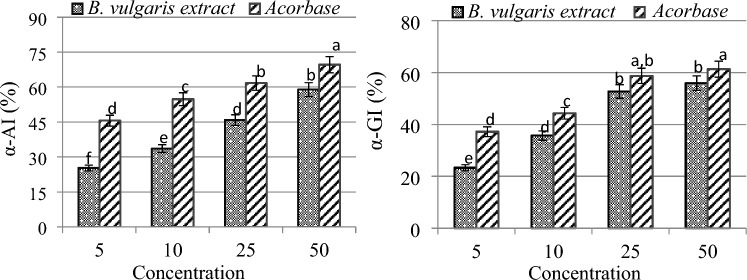
Effects of *B. vulgaris* extract on α-amylase and α-glucosidase activity. *B. vulgaris* extract (mg/mL), Acorbase (µg/mL). α-AI: alpha amylase inhibition, α-GI: alpha glucosidase inhibition. Means shown with different letters^(a–e)^ are statistically significant (*p* < 0.05).

Anti-diabetic activity of *B. vulgaris* extract was determined by measuring inhibitions of α-amylase and α-glucosidase, and the results are given in Fig. 7. While 5–50 mg/mL *B.vulgaris* extract inhibited α-amylase activity between 25.3 and 58.9%, it caused inhibition in α-glucosidase activity between 23.3 and 55.9%. The enzyme inhibition effect of the extract increased in a dose-dependent manner. 5–50 µg/mL acorbase, used as a standard substance, inhibited α-amylase in the range of 46.6% and 69.6%, and α-glucosidase between 37.2 and 61.3%. *Beta vulgaris* extract showed a higher inhibitory effect on α-glucosidase enzyme compared to α-amylase, while acarbose showed a higher effect on α-amylase. The literature has documented the presence of over 400 plant species with anti-diabetic properties; nevertheless, the diversity of these species and their varying phytochemical composition under various ecological settings render these researches inadequate. Additionally, plants with anti-hyperglycemic properties work through a variety of methods, including enhancing tissue uptake of glucose, decreasing intestinal absorption of glucose, and inhibiting the synthesis of glucose in the liver. Reduced digestion of dietary carbohydrates is the primary mechanism by which plants exert their anti-diabetic effects. In particular, the anti-diabetic activity of herbal extracts is achieved by the inhibition of α-glucosidase and α-amylase enzymes. In this way, the conversion of dietary carbohydrates to glucose and the sudden increase in blood glucose level can be reduced^59,60^. The inhibitory effect of *B. vulgaris* extract on both enzymes indicates its anti-hyperglycemic effect and its ability to reduce the risk of diabetes. This feature can be explained by the biological roles of the active ingredients in the extract. Phytol is a phytochemical with potent anti-diabetic properties. Sulaimon et al.^61^ reported that *Piper guineense* extract, which was found to contain major phytonutrients, has strong anti-diabetic activity. Şen et al.^62^ reported the anti-hyperglycemic activity of *Centaurea pterocaula* extract by inhibiting α-amylase activity, and they associated this activity with essential oil components such as hexahydrofarnesyl acetone detected in the extract content.

In this study, the anti-microbial, anti-mutagenic and anti-proliferative activities of the extract were investigated by in-vivo systems. Since the anti-diabetic activity was determined by in-vitro methods, enzyme inhibitions were investigated by molecular docking to support the results. For this purpose, in-silico interactions of α-amylase and α-glucosidase enzymes with major contents of extract were investigated.

### Molecular docking

**Figure 8:**
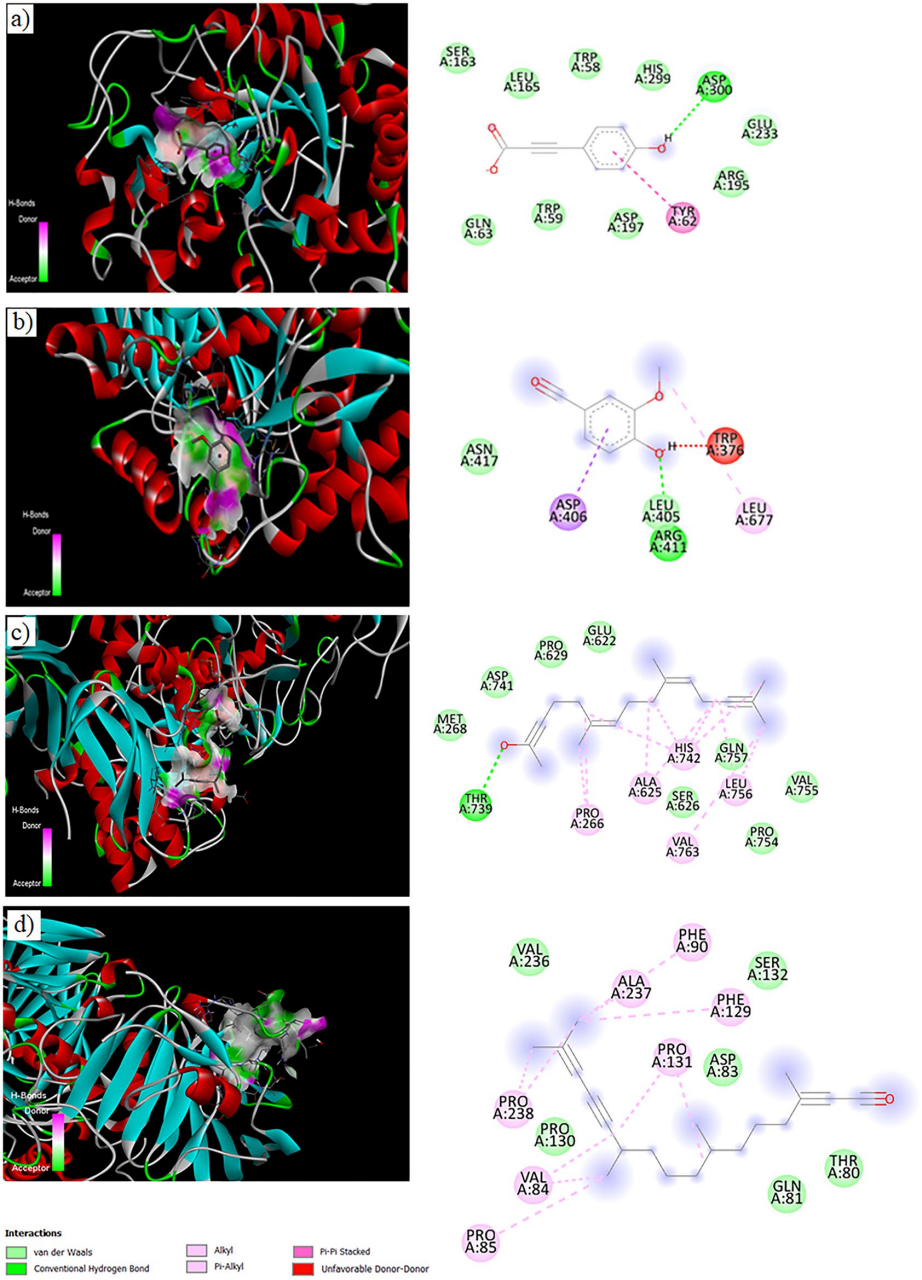
Molecular docking of α-glucosidase with p-coumaric acid (**a**), vannilin (**b**), hexahydrofarnesyl acetone (**c**), phytol (**d**).

**Figure 9:**
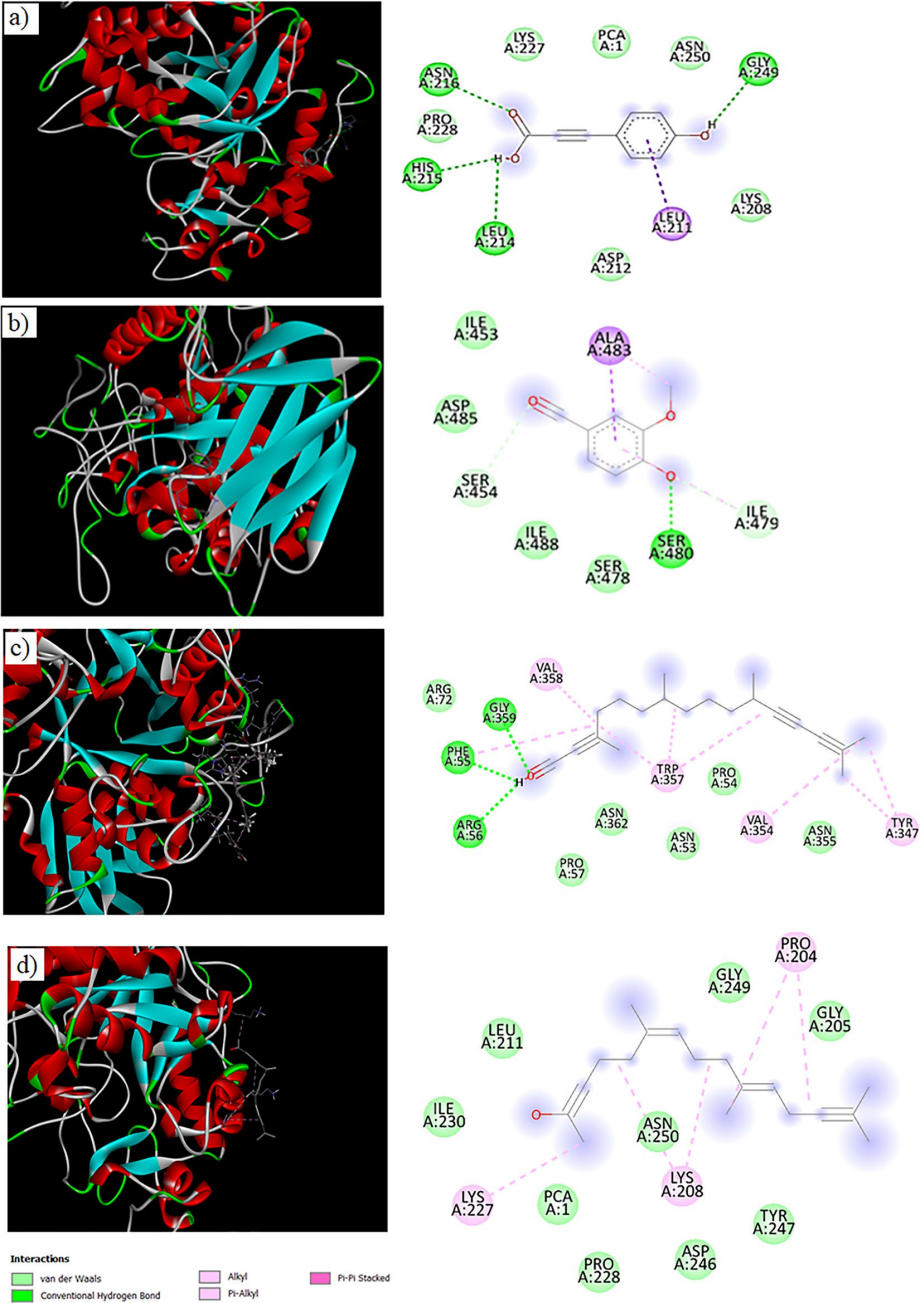
Molecular docking of α-amylase with p-coumaric acid (**a**), vannilin (**b**), phytol (**c**), hexahydrofarnesyl acetone (**d**).

Enzyme inhibitions indicating the anti-hyperglycemic effect of *B. vulgaris* were confirmed in-silico, and for this purpose, the interactions of α-amylase and α-glucosidase enzymes with the major compounds found in the extract were investigated. The interactions of p-coumaric acid and vannilin detected in LC–MS/MS, hexahydrofarnesyl acetone and phytol detected in GC–MS analysis were investigated. α-amylase showed a binding energy in the range of − 4.3 kcal/mol and − 6.1 kcal/mol with the tested active compounds. In the α-glucosidase, this range was determined as − 3.7 kcal/mol and − 5.7 kcal/mol (Table 6). Each compound interacted with α-amylase and α-glucosidase enzymes through different amino acids (Figs. 8 and 9). It has been determined that vander-waals and hydrogen bonds are common bonds observed in all interactions. Some other interactions such as Pi–sigma (coumaric acid–α-amylase), unfavorable donor–donor (vanillin–glucosidase) or Pi–Pi (coumaric acid– α-glucosidase) were also observed. These changes observed in the bonds formed during interactions can be associated with the different amino acid content of each enzyme and the accessibility of amino acids. The interaction of enzymes with the tested compounds can cause changes in the three-dimensional structure and loss of function, namely inhibition. Inhibition of these enzymes also leads to a decrease in the digestion of dietary carbohydrates and the passage of glucose into the blood, thus providing an anti-diabetic effect. Similarly, Akshatha et al.^63^ reported that the active compounds of *Leucas ciliata* such as topotecan, cathine, lucenin, interact with the α-amylase enzyme through bonds such as hydrogen, Pi–sigma, Pi–alkyl, and that an anti-diabetic effect can occur in this way. Molecular docking is used in many literature studies to confirm in-vitro test results^64–69^. In this study, inhibitions of α-amylase and α-glucosidase detected by in-vitro analyses were supported by in-silico study and the anti-diabetic activity of *B. vulgaris* was confirmed.

**Table 6:** Molecular docking datas of α-amylase and α-glucosidase interactions.

Enzyme	Compound	Binding energy	Interacted aminoacid
α-glucosidase	p_coumaric acid	− 5.7 kcal/mol	GLU, SER, LEU, TRP, HIS, ASP,ARG, TYR, ASP, GLN
	Vanillin	− 4.8 kcal/mol	ASP, ARG, ASN, LEU, TRP
	Hexahydrofarnesyl Acetone	− 3.7 kcal/mol	GLU, PRO, ASP, MET, THR, ALA, HIS, GLN, LEU, VAL
	Phytol	− 4.3 kcal/mol	VAL, PHE, ALA, SER, PRO, ASP, THR, GLN
α-amylase	p_coumaric acid	− 6.1 kcal/mol	ASN, PRO, HIS, LEU, ASP, LYS, GLY
	Vanillin	− 5.1 kcal/mol	ILE, ALA, ASP, SER
	Hexahydrofarnesyl Acetone	− 4.3 kcal/mol	LEU, GLY, PRO, ASN, LYS, TYR, ILE
	Phytol	− 4.6 kcal/mol	ARG, GLY, TRP, PHE, VAL, ASN, PRO

## Conclusion

Plants are natural foods with many biological and pharmacological activities. Herbal bioactive compounds exhibit positive effects on health when consumed as food and attract more attention compared to synthetic materials in the treatment of chronic diseases. In this study, the phytochemical fingerprint and biological activities of *B. vulgaris* extract, which is consumed as food in Turkiye and many countries, were investigated. While high levels of betalain and betacyanin were detected in the extract content, the presence of protecatechuic aldehyde, sesamol, vannilin and p_coumaric acid was determined as phenolic compounds. In the extract containing a wide variety of essential oils, hexahydrofarnesyl acetone and phytol were determined as major essential oils. As a result, the extract has a broad biological activity profile that does not show cytotoxic effects, has anti-mutagenic, anti-proliferative and anti-microbial activity. *Beta vulgaris* exhibited broad-spectrum antimicrobial activity, and the highest inhibition was obtained against *S. aureus*, a gram-positive bacteria. The extract, which exhibited significant anti-mutagenic activity by reducing chromosomal abnormalities in the range of 35.9–61.8%, reduced α-amylase activity by 46.6–69.6% and α-glucosidase activity by 37.2–61.3%. The anti-hyperglycemic activity of *B. vulgaris* extract may be due to the active ingredients it contains and the synergistic effects of these ingredients. The chemical components of plant extracts can help reduce the risk of developing various diseases such as diabetes and can serve as an alternative or supplement to existing drug applications. Cytotoxic or mutagenic effects of herbal extracts should also be investigated for safer use against diseases. This study shows that *B. vulgaris* extract, which is not cytotoxic and mutagenic and exhibits anti-mutagenic activity, can be used for anti-hyperglycemic purposes. The chemical components of plant extracts can help reduce the risk of developing various diseases such as diabetes and can serve as an alternative or supplement to existing drug applications. Herbal products exhibiting biological activity must be standardized before being offered for drug or medical use, and their effectiveness and reliability on humans must be proven by scientific methods.

## Data Availability

All data generated or analyzed during this study are included in this published article.

## Abbreviations

BC: Betacyanin
BX: Betaxanthin
CAs: Chromosomal abnormalities
EE: Extraction efficiency
GAE: Gallic acid equivalent
GC–MS: Gas chromatography–mass spectrometry
LC–MS/MS: Liquid chromatography mass spectrometer
MIC: Minimum inhibitory concentration
TPC: Total phenolic content
